# Females' Knowledge and Use of Isotretinoin (Roaccutane) in the Western Region of Saudi Arabia

**DOI:** 10.7759/cureus.12148

**Published:** 2020-12-18

**Authors:** Kholoud Mohammed A Bakheet, Rand G Alghanemi, Alya M Alsiyoufi, Mohammed Abduljabbar, Jehad Hariri

**Affiliations:** 1 Dermatology, King Abdulaziz University, Jeddah, SAU; 2 Dermatology, King Abdulaziz University Hospital, Jeddah, SAU

**Keywords:** roaccutane, isotretinoin, acne, knowledge, practice

## Abstract

Background

Acne vulgaris is one of the most common diseases worldwide. It is a chronic, relapsing inflammatory skin disease. Nearly anyone can be affected at any age. Adolescents and young adults are more susceptible, with a prevalence as high as 35% to 90% and reaching up to 100% in both sexes. Isotretinoin is the most effective medication to be used. It has been reported in the literature that many populations are non-adherents to or aware of safety recommendations. This study aims to assess females’ awareness and safety of isotretinoin use in the western region of Saudi Arabia.

Methods

This is a cross-sectional, descriptive study. A semi-structured questionnaire was used, data was collected from an electronic validated survey and published on a social platform. Statistical analysis was conducted with the aid of Statistical Package for Social Sciences (SPSS) version 21 (IBM Corp., Armonk, NY, USA).

Results

The total number of included responses was 1066. Most of the participants were 12-22 years old (45.2%), single (72.2%) and had a bachelor’s degree (69.6%). Among the total number of participants there were 285 participants who used isotretinoin. Ninety-three percent of them had a prescription of isotretinoin from a physician. The common dose given was approximately 20 mg and the common duration was more than six months.

Conclusion

There is a good amount of knowledge in our population regarding isotretinoin side effects, although only half of them were informed about them by their treating physicians. We noticed an obvious lack of awareness about safe practice during childbearing age and marriage. This highlights the need for optimal education by health care providers and the role of media is obvious to improve their practices and hopefully decrease the risks as it was the second most common source of information after dermatologists in our population. Also we recommend paying more attention to the psychological side effect which was reported by 9% of our participants.

## Introduction

Acne vulgaris is one of the most common diseases worldwide [1]. It is a chronic, relapsing inflammatory skin disease. Adolescents and young adults are more susceptible, with a prevalence as high as 35% to 90% and reaching up to 100% [2]. It was also reported among adults in their 50s and above in both sexes [3]. In 2016, more than 50% of adolescent males in the southern region of Saudi Arabia were found to have acne [4]. An epidemiological study was conducted in 2019 in Jeddah, Saudi Arabia; this showed that around 60% of female medical students at King Abdulaziz University are suffering from acne. The high prevalence of the disease is thought to be multifactorial and caused by various factors such as hormones, stress, unhealthy lifestyle, anxiety, and depression [5]. In addition, we should pay attention to its negative effect on self-esteem, body image, and quality of life, especially among females. Regarding treatment guidelines, different medications were approved for use according to disease severity. Isotretinoin is the most effective treatment for severe, resistant cases [6]. It carries many unfavorable side effects such as teratogenicity, abnormal lipid profile, psychiatric issues, and dryness, which is the most common complaint among users [7]. Due to its teratogenicity, females of childbearing age must be advised to be on two or more contraception methods while on treatment [8]. In addition, regular monitoring and patient follow-up are recommended. This highlights the importance of proper patient knowledge and education by their dermatologists before starting treatment. A 2018 study to assess the public understanding and awareness of isotretinoin was conducted in Al Ahsa, and it showed that only half of the participants were able to identify most of the side effects; this was indicative that there was a lack of knowledge regarding the use of isotretinoin among the study population [9]. Moreover, another study among females students at Al-Faisal University was conducted in 2019; this study showed that acne was prevalent in 76.9% of the sample, with 48.6% of them using isotretinoin. Of that 48.6% however, 3.2% were self-prescribing isotretinoin, which is against the recommended guidelines. A total of 81.9% of users could correctly identify the side effects of the medication. However, the knowledge about double contraception use among women of childbearing age was deficient [10]. To the best of our knowledge, no similar previous research was done among females in our local population. Therefore, we aimed to conduct a cross-sectional study to assess the knowledge and safety of isotretinoin use in the western region of Saudi Arabia.

## Materials and methods

This cross-sectional, descriptive study was reviewed by the scientific board, and ethical approval was obtained from King Abdulaziz University Hospital, Jeddah, Saudi Arabia. A total of 1257 responses were collected, using an electronic validated Arabic questionnaire taken with permission from the authors of a previous similar study [11]. To increase our sample size, the questionnaire was disseminated through social media (Whats-App and Twitter). Men and women from regions other than the western area were excluded. Several sections, besides socio-demographic sections, were included in the questionnaire. All participants were asked to choose all known side effects of isotretinoin, with correct and incorrect answers. Additionally, users were asked to choose the side effects that they experienced, and if they used any contraception methods while on treatment. Statistical analysis was conducted with Statistical Package for Social Sciences (SPSS) version 21 (IBM Corp., Armonk, NY, USA).

## Results

In this study, 1257 female participants were recruited; 191 of which were found not to be from the Western region of Saudi Arabia and were therefore excluded from the study. Table 1 shows the demographic characteristics of the study population. It shows that 85.4% of the population were Saudi Arabian, and the majority (82.7%) were from Jeddah. Most of the participants were single (72.2%) and had a bachelor’s degree (69.6%). Participants were categorized into three age groups: 12-22 years (45.2%), 23-33 years (37.8%), and 34-60 years (17%).

**Table 1:** Demographic characteristics of the participants (n=1066).

Demographics	N (%)
Age group	
12-22	482 (45.2)
23-33	403 (37.8)
34-60	181 (17)
Region	
Jeddah	882 (82.7)
Makkah	78 (7.3)
Al-Madinah	66 (6.2)
Taif	14 (1.3)
Yunbu	19 (1.8)
Rabegh	7 (0.7)
Marital status	
Single	770 (72.2)
Married	266 (25)
Divorced / Widowed	30 (2.8)
Nationality	
Saudi	910 (85.4)
Non-Saudi	156 (14.6)
Education level	
Middle school	11 (1)
High school	219 (20.5)
Diploma	33 (3.1)
Bachelor's degree	742 (69.6)
Master / Doctoral	61 (5.7)

Table 2 represents the questions asked to the 285 participants who used isotretinoin. It shows that 93% of the participants had a prescription for isotretinoin from a physician. The common dose given was approximately 20 mg, with 29.8% of the participants taking this amount. The duration of treatment was divided into two groups: less than six months (46%) and more than six months (54%). Regarding the side effects that were experienced, patients were allowed to choose multiple side effects, where dryness was the most experienced side effect (80.7%). On the other hand, depression and suicidal thoughts were the least experienced side effects (8.8%). Approximately 84.6% of respondents were informed about the side effects of isotretinoin from different sources, 51.6% of which were informed by a physician verbally most of the time (85%).

**Table 2:** Users of isotretinoin experience (n=285) *Respondents may choose more than one answer to this question.

Isotretinoin users questions	Answers	N (%)
Who prescribed Isotretinoin?	Doctor	265 (93)
	Pharmacist	3 (1)
	Self-medicated	5 (1.8)
	Family/Friends	12 (4.2)
Dose (mg)	10	17 (6)
	20	85 (29.8)
	30	47 (16.5)
	40	41 (14.4)
	50	8 (2.8)
	I don’t remember	87 (30.5)
Duration of treatment (months)	<6	131 (46)
	>6	154(54)
Side effects experienced*	No side effects	29 (10.2)
	Dryness	230 (80.7)
	Depression and suicidal thoughts	25 (8.8)
	Cholesterol and triglycerides	27 (9.5)
	Musculoskeletal pain	60 (21.1)
	Hair loss	78 (27.4)
	Miscellaneous	46 (16.1)
Were you informed about the side effects of Isotretinoin?	Yes	241 (84.6)
	No	44 (15.4)
Who informed you about the side effects of the Isotretinoin?	Doctor	147 (51.6)
	Pharmacist	6 (2.1)
	Nobody informed me	44 (15.4)
	Family/Friends	30 (10.5)
	Social media	58 (20.4)
If you have been informed by a physician or a pharmacist, how did that happen?	Verbally	130 (85)
	Written	4 (2.6)
	Both	19 (12.4)

The relationship between the awareness of the side effects of isotretinoin and using isotretinoin is shown in Table 3. Participants were asked to choose from a list of side effects that they were aware of. In the list, half of the side effects were correct (e.g., dryness, depression, liver injury, congenital malformation, miscarriage, and increased blood cholesterol), whereas the other half was incorrect (e.g., increased blood glucose, renal failure, and sleep disorders).There was a significant difference between the use of isotretinoin and being unaware of its side effects, as non-users were more unaware of the side effects of the drug compared to users (p=<0.001). Acknowledging the correct side effects and use of isotretinoin showed a statistically significant difference with a p-value of <0.001. Regarding the incorrect side effects, no statistical differences were seen between users and non-users regarding increased blood glucose levels and occurrence of sleep disorders. However, in comparison to users (p<0.001), non-users were more likely to say that miscarriage and renal failure were not side effects of isotretinoin.

**Table 3:** Awareness of isotretinoin among users and non-users.

Side effects	Users n(%)	Non-users n(%)	P-value
I don’t know anything:			<0.001
Yes	17(6)	174(22.3)	
No	268(94)	607(77.7)	
Correct side effects:			
1- Liver injury			<0.001
Yes	180(63.2)	328(42)	
No	105(36.8)	453(58)	
2- Increased blood cholesterol			<0.001
Yes	111(38.9)	86(11)	
No	174(61.1)	695(89)	
3- Congenital malformation			<0.001
Yes	189(66.3)	288(36.9)	
No	(33.7)96	(63.1)493	
4- Depression			<0.001
Yes	(50.2)143	(28.4)222	
No	(49.8)142	(71.6)559	
5- Dryness			<0.001
Yes	252(88.4)	529(67.7)	
No	(11.6)33	252(32.3)	
6- Miscarriage			<0.001
Yes	102(35.8)	165(21.1)	
No	183(64.2)	616(78.9)	
Incorrect side effects:			
1- Increased blood glucose			0.71
Yes	15(5.3)	35(4.5)	
No	270(94.7)	746(95.5)	
2- Renal failure			<0.001
Yes	91(31.9)	169(21.6)	
No	194(68.1)	612(78.4)	
3- Sleep disorders			0.49
Yes	38(13.3)	90(11.5)	
No	247(86.7)	691(88.5)	

In Table 4, statistical analyses were made to assess the relationship between the duration of treatment and the side effects experienced. It was concluded that patients who used isotretinoin for less than six months did not experience side effects compared to patients who used it for more than six months (p=<0.001). In addition, dryness and hair loss were found to be more commonly experienced by patients who used it for more than six months (p-value <0.001). 

**Table 4:** Side effects in relation to the duration of treatment. *MSK: musculoskeletal

Side effects	<6 month n(%)	>6 month n(%)	P-value
Nothing			<0.001
Yes	22(16.8)	7(4.5)	
No	109(83.2)	147(95.5)	
Depression			0.21
Yes	8(6.1)	17(11)	
No	123(93.9)	137(89)	
Dryness			<0.001
Yes	91(69.5)	139(90.3)	
No	40(30.5)	15(9.7)	
Increased cholesterol level			0.71
Yes	11((8.4)	16(10.4)	
No	120(91.6)	138(89.6)	
Hair loss			<0.001
Yes	24(18.3)	54(35.1)	
No	107(81.7)	100(64.9)	
MSK* pain			0.37
Yes	24(18.3)	36(23.4)	
No	107(81.7)	118(76.6)	
Miscellaneous			0.24
Yes	17(13)	29(18.8)	
No	114(87)	125(81.2)	

Another interrelation between the side effects experienced and the dose of isotretinoin given was studied. It was found that almost none of them had statistical differences except for musculoskeletal pain, which showed a significant difference with the dose given (p=0.041). Moreover, awareness of isotretinoin side effects (p=0.005), its use (p=0.002), and the current presence of acne (p<0.001) showed a statistically significant relationship with the age group that the patient belonged to.

Out of 285 participants who used isotretinoin, 22 used it after marriage. Most of them (77.3%) were advised to use one method of contraception, while two (9.1%) said that no prescription was prescribed to them. Ninety percent were advised by a doctor to use contraception during treatment. Out of the 22 married females who took isotretinoin, 20 knew about congenital malformations. Seventeen of them were told by their doctors, four read about it, and two had not been told. Moreover, six of the participants underwent a pregnancy test before starting the treatment, while three took a pregnancy test during the treatment. Only two participants became pregnant while using the drug.

## Discussion

There have been many treatments used for acne. However, unlike other treatments, isotretinoin revolutionized acne treatment by treating all the known primary etiologies of acne [12].

In the present study, we focused on assessing female knowledge, safety awareness, and use of isotretinoin. Of our 1066 participants, 285 females had previously used isotretinoin; 93% of which had used it based on a prescription of a physician. The possession of a prescription for isotretinoin was expected, knowing that a prescription and consent is needed in order for a pharmacy to dispense it. This study is also within the range of two previous studies [10,11].

We found that isotretinoin users had better awareness of the possible side effects of the treatment, unlike non-users (see Table 3), with increased blood cholesterol being the least acknowledged side effect among users (38%), which is consistent with what was found in another Saudi study [10]. 

When it comes to experiencing side effects, most patients experienced skin dryness (80%). On the other hand, the least experienced side effects were depression and suicidal thoughts (8.8%); this finding is consistent with what was found in previous studies [13-15].

Moreover, patients receiving treatment for more than six months were significantly more likely to experience side effects than patients receiving treatment for less than six months; those receiving higher dosing did not experience more side effects, which is in line with two studies that found no statistical difference in cumulative dosing and side effect occurrence [16-17].

Assuming that sexual activity only happened after marriage due to our conservative society, we asked about the use of isotretinoin after marriage (married participants were a group of 22) and the use of contraception; it was found that 20 out of 22 participants were well aware of the teratogenicity that might occur if they became pregnant while on isotretinoin since 90% were advised by a doctor to use contraceptive measures. However, their knowledge appeared to be incomplete regarding the proper numbers of contraception as per guidelines. In addition, two of the participants became pregnant while on medication. These results coincide with another Saudi study, which revealed the need to increase proper awareness of contraception usage among sexually active groups in our society [12]. Furthermore, a Saudi study conducted in Riyadh in 2011 found that only 62% of dermatologists advised the need for using two contraception methods while on isotretinoin [14]; another study in Riyadh found the need for improvement when it comes to married females on isotretinoin risk reduction counseling skills [18].

To the extent of our knowledge, no previous similar studies have been done among females in the western region of Saudi Arabia, although this is a cross-sectional study through an online questionnaire with possible recall bias.

## Conclusions

In conclusion, our population showed good knowledge regarding the side effects of isotretinoin. However, only half of them were informed by their treating physicians, and 15% had not been told about these side effects at all. Isotretinoin users identified most of the side effects more correctly than non-users. We noticed that female practice recommends the use of contraception, pregnancy testing, and isotretinoin (use of which) was deficient, which was reflected in the two cases wherein the women got pregnant. Therefore, we recommend that their dermatologists help increase awareness and correctly educate them about this important issue before starting its use. Moreover, we would like to highlight that around 9% of users complain of depression and suicidal thoughts; although these were the least common side effects experienced, we recommend paying more attention to this psychological aspect.
